# A retrospective study of pregnant patients with acute pancreatitis

**DOI:** 10.1590/1806-9282.20230810

**Published:** 2024-03-15

**Authors:** Şehmus Ölmez, Bünyamin Sarıtaş, Mehmet Suat Yalçın, Raziye Narin, Adnan Taş, Nevin Akçaer Öztürk, Mustafa Muslu, Haşim Nar, Ekrem Sapmaz, Banu Kara

**Affiliations:** 1 University of Health Sciences, Adana City Training and Research Hospital, Department of Gastroenterology – Adana, Turkey.; 2 Muğla Training and Research Hospital, Department of Gastroenterology – Muğla, Turkey.; 3 University of Health Sciences, Adana City Training and Research Hospital, Department of Gynecology and Obstetrics – Adana, Turkey.

**Keywords:** Pancreatitis, Pregnancy, Prognosis

## Abstract

**OBJECTIVE:**

Acute pancreatitis is a rare disease in pregnant patients. Although it may have serious maternal and fetal consequences, morbidity and mortality rates have decreased recently due to appropriate and rapid treatment with earlier diagnosis. The aim of this study was to evaluate pregnant patients diagnosed with acute pancreatitis.

**METHODS:**

The study included pregnant patients diagnosed with acute pancreatitis who were admitted to Adana City Training and Research Hospital in Adana, Turkey, between January 2014 and January 2022. Patients’ files were screened. Patients’ demographics, acute pancreatitis etiology, severity, complications, and applied treatment, as well as maternal and fetal outcomes were evaluated.

**RESULTS:**

The study included 65 pregnant patients with acute pancreatitis. The mean age was 26.6±5 (19–41) years. Acute pancreatitis was observed in the third trimester. The most common cause of acute pancreatitis was gallstones, and its severity was often mild. Only two patients required endoscopic retrograde cholangiopancreatography, and the remaining patients were treated medically. Maternal and infant death developed in a patient with necrotizing acute pancreatitis secondary to hyperlipidemia.

**CONCLUSION:**

The most common etiology of acute pancreatitis in pregnancy was gallstones. Acute pancreatitis occurred in the third trimester. Most of the patients had mild acute pancreatitis. Maternal and fetal complications were rare. We think that the reasons for the low mortality rate were mild disease severity and biliary etiology, and most patients were in the third trimester, as well as early diagnosis and no delay in the intervention.

## INTRODUCTION

Acute pancreatitis during pregnancy (APDP) is a rare disease. The incidence of APDP varies and is 1/1000 to 3/10000 in pregnancies^1,2^. The prevalence of APDP is not different in nonpregnant patients^3^. The most common cause of APDP was gallstone, accounting for 36.4–70% of all cases^1,4,5^. The second-most common cause of acute pancreatitis (AP) is hyperlipidemia^6,7^. APDP caused by hyperlipidemia is associated with more maternal and fetal undesirable complications^4,6,7^. Recent studies have shown that the prognosis for APDP is not different from that of nonpregnant patients^8^. Due to improvements in both in diagnostic modalities and care in intensive care units and neonatal intensive care units, maternal and fetal mortality related to AP has decreased recently^1,4,6^. However, the development of AP in pregnant patients leads to serious stress in both patients and their relatives.

Acute pancreatitis is an important problem in gastroenterology clinical practice^9^. The most common causes of AP are gallstones, followed by hyperlipidemia and alcohol^10^. The clinical course of AP is classified as mild, moderate, and severe. In general, AP has a mild course, but it may have a severe course, leading to pancreatic necrosis, abscess, or organ dysfunctions even to death^9^.

There are guidelines about the management of AP^9,11-13^. However, there is no guideline for the diagnosis and treatment of APDP. Since AP is rarely seen in pregnant patients and information about clinical follow-up and treatment is uncertain^3,4^, it is very important to develop proper diagnostic algorithms and treatment strategies^5^. Besides, accompanying cholelithiasis and choledocholithiasis may affect the proper treatment choice and timing of treatment modalities such as endoscopic retrograde cholangiopancreatography (ERCP) or surgery. Patients must be followed up by a multidisciplinary approach, including gastroenterologists and obstetricians^5^.

In this study, we aimed to evaluate patients with APDP, their treatment and prognosis, as well as maternal and fetal outcomes.

## METHODS

Pregnant patients diagnosed with AP admitted to the gastroenterology department of Adana City Training and Research Hospital (Adana, Turkey) during the period of January 2014–January 2022 were included in the study. Patients’ files and hospital computer databases were screened retrospectively. Patients’ demographics, etiology of AP, and clinical and laboratory data were recorded. The duration of both hospital stay and intensive care stay, medical treatment records, maternal and fetal outcomes, and complications were recorded.

The diagnosis of AP and severity were made according to Atlanta criteria. The diagnosis of AP was made if two out of three criteria existed: 1. Abdominal pain (typical abdominal pain of AP is acute epigastric pain spreading to the back) 2. Above three times the upper limit of normal amylase and lipase levels. 3. Diagnostic imaging consistent with AP [computed tomography (CT), magnetic resonance imaging (MRI), or transabdominal ultrasonography (USG)]. Severity of AP was classified as mild if there were no signs of organ failure besides no local or systemic complications; moderate if there was transient organ failure (relieving in 48 h) and/or local or systemic complications without persistent organ failure (>48 h); or severe if there was persistent one or more organ failure according to revised Atlanta criteria^14^. Also, modified Ranson and modified Glasgow scores were also calculated to define the severity of AP^15,16^.

Patients were categorized according to age. Patients’ gestational age was determined according to the following: the first trimester was defined as weeks 1–13, the second trimester as weeks 14–27, and the third trimester as 28 weeks or longer gestational week.

### Statistics

Statistical analysis was made with Statistical Package for the Social Sciences (SPSS) version 23 (IBM Inc.). Continuous variables are explained as mean±standard deviation (SD) (min–max), and categorical variables are given as frequency and percentage [n (%)].

## RESULTS

**Figure 1:**
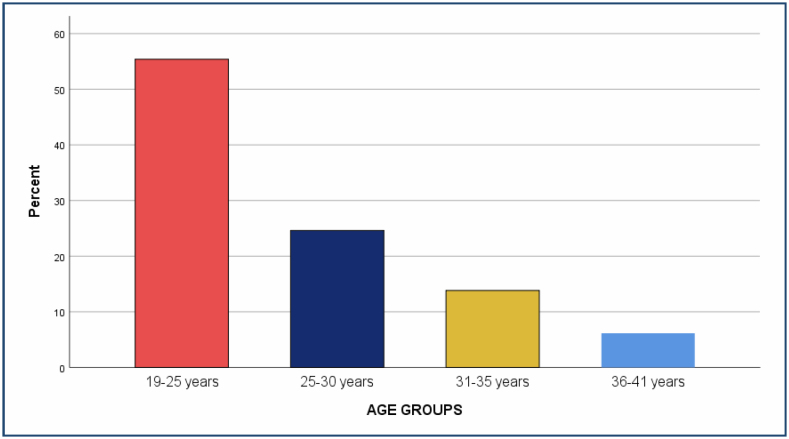
Distribution of patients according to age groups.

A total of 65 patients were included in the study. The mean age was 26.42±5 (19–41) years. Of note, 36 (55.4%) patients were 19–25 years old, 16 (24.6%) patients were 26–30 years old, 9 patients were 31–35 years old, and 4 (6.2%) patients were 36–41 years old (Figure 1). The mean number of gravida was 2.75±2.1 (1–10), parity was 1.2857±1.58 (0–5), and abortus was 0.8276±1.41595 (0–5). Pancreatitis was observed in the third and second trimesters, respectively.

The most common etiologies of pancreatitis were biliary origin and hyperlipidemia. One patient had post-ERCP pancreatitis. Most of our patients [61 (93.8%)] had mild pancreatitis. The Ranson score and modified Glasgow score of our patients were 0.59±1.2 (0–6) and 0.53±0.76 (0–3), respectively. The mean duration of hospital stay was 4.3±2.5 (1–14) days for service and 0.9±3.9 (0–27) days for intensive care. Most of the patients were treated conservatively. Only two patients (3.1%) required ERCP. Only one patient with necrotizing pancreatitis secondary to hyperlipidemia required lipid apheresis, and maternal and infant death developed in that patient despite lipid apheresis. The patient was 30 years old, and had one parity with a healthy child and a history of AP. She died of AP related to hyperlipidemia during her second pregnancy. One patient had an early delivery at 37 weeks. Patients’ demographic data, gravida and parity status, etiology, severity of pancreatitis, applied treatment modalities, and prognosis are summarized in Table 1. Patients’ laboratory data are summarized in Table 2.

**Table 1:** Demographic and clinical information of patients.

Age (years) (mean±SD) (min–max)		26.42±5 (19–41)
Gravida		2.75±2.1 (1–10)
Parity		1.2857±1.58
**Trimester of pregnancy**		n (%)
	First	11 (16.9%)
	Second	15 (23.1%)
	Third	39 (60%)
**Etiology**		n (%)
	Biliary	50 (76.9%)
	Hyperlipidemia	7 (10.8%)
	Post-ERCP	1 (1.5%)
	Idiopathic	7 (10.8%)
**Severity of acute pancreatitis**		n (%)
	Mild	61 (93.8%)
	Moderate	1 (1.5%)
	Severe	3 (4.6%)
Ranson score		0.59±1.2 (0–6)
Modified Glasgow score		0.53±0.76 (0–3)
Duration of ICU stay (days)		0.9±3.9 (0–27)
Duration of inpatient follow-up (days)		4.3±2.5 (1–14)
Total duration of hospital stay (days)		5.1±4.7 (1–28)
**Treatment**		n (%)
	Medical	63 (96.9%)
	ERCP	2 (3.1%)
**Prognosis**		n (%)
	Maternal mortality	1 (1.5%)
	Fetal mortality	1 (1.5%)

ERCP: endoscopic retrograde cholangiopancreatography; SD: standard deviation; ICU: intensive care unit.

**Table 2:** Patients’ laboratory data on admission.

	Mean±SD (min–max)
WBC (/μL)	12220±4053 (6900–27000)
Hb (g/dL)	11.3±1.4 (8.1–15.6)
CRP (mg/L)	13.2±23.9 (0.1–143)
Glucose (mg/dL)	106.3±26.5 (68–208)
AST (U/L)	92.8±126.6 (10–942)
ALT (U/L)	74.2±111.8 (3–497)
Alb (g/L)	35.4±4.4 (24.6–48.1)
T bil (mg/dL)	1.2±1.1 (0.2–5.2)
ALP (U/L)	146.9±87 (43–469)
GGT(U/L)	73.8±78.8 (6–424)
LDH (U/L)	297.8±172.8 (136–1175)
Ca (mg/dL)	8.8±0.6 (7.3–10.1)
BUN (mg/dL)	15.7±5.6 (4–32)
Cr (mg/dL)	0.43±0.12 (0.16–0.87)
NA (mmol/L)	135.6±4.4 (123–143)
K (mmol/L)	4.4±0.5 (3.1–6)

WBCs: white blood cells; Hb: hemoglobin; CRP: C-reactive protein; Alb: albumin; AST: aspartate aminotransferase; ALT: alanine aminotransferase; T Bil: total bilirubin; ALP: alkaline phosphatase; GGT: gamma-glutamyl transferase; LDH: lactic dehydrogenase; BUN: blood urea nitrogen; Cr: creatinine; Na: sodium; K: potassium.

## DISCUSSION

Although AP is a rare disease in pregnant women, the severity and etiology of pancreatitis should be determined to diagnose and treat these patients earlier, since it may have serious consequences in both the mother and the fetus^1,5,8^. Determining the exact trimester is also important to choose the correct treatment modality. Obstetricians should also evaluate the status of fetus at the beginning and when necessary. Determining the etiology of pancreatitis is very important since the treatment choice of ERCP, timing of cholecystectomy, or dietary modification affect the prevention of pancreatitis^6,9^.

Pancreatitis in pregnant patients is diagnosed by using abdominal USG, abdominal CT, MRI, and endoscopic ultrasonography (EUS)^6^. In the selection of imaging method, the potential risks on the fetus should also be considered. Abdominal USG is the first diagnostic choice in the diagnosis and etiology of APDP. It is noninvasive, cost-effective, and safe. But its diagnostic capacity is restricted, and it depends on the operator's experience, obesity, and intestinal gas. While the sensitivity of USG is good for cholelithiasis, it is poor for choledocholithiasis and pancreatitis^6,17^. Abdominal CT is commonly used in the diagnosis of AP, both in diagnosis and determining the severity of pancreatitis, but in pregnant patients its use is limited due to the potential risk of ionizing radiation and contrast agents on the fetus. So, it is not recommended in the APDP^6,17^. Magnetic resonance cholangiopancreatography (MRCP) is a very effective diagnostic modality in pregnant patients because it does not have ionizing radiation and contrast agents, and it is also very sensitive in diagnosing choledocholithiasis. EUS is also effective in diagnosing biliary stones and sludge, but it cannot be done in every case. It is performed under anesthesia. This method may be useful in patients for whom a high probability of choledocholithiasis is suspected but an abdominal USG or MRCP shows no biliary stone. It may be done prior to ERCP to prevent unnecessary ERCP procedures^17^.

The etiology of APDP is similar in both pregnant and nonpregnant patients, as biliary stones are the most common etiology^1,2,7^. In normal pregnancy, some physiological changes occur in women. There is an increase in gallbladder volume, and the bile flow slows down. The most common contributing factors to these changes are increased estrogen and progesterone hormone levels^6^. In pregnancy, gallbladder stones and the frequency of biliary pancreatitis are increased^18^. Hyperlipidemia is another leading contributing factor to AP^7,8,19^. Other causes are drugs, trauma, pregnancy-induced hypertension, acute fatty liver disease of pregnancy, and genetic disorders. Idiopathic pancreatitis may also be observed^7,20^. Although alcoholic pancreatitis is frequently seen in the etiology of AP in nonpregnant patients, it is very rare in pregnant women. In some studies, it is not reported in the etiology of APDP^3,4,7,21^. Most studies reported that APDP was observed in the third trimester, and the most common cause was gallstone^6,19^. In our study, the most common etiologic factors were biliary stone disease (76.9%) and hyperlipidemia (10.8%). One patient had post-ERCP pancreatitis, for which ERCP was performed for choledocholithiasis. We have no cases related to alcohol. We have identified no etiologic factors in 10.8% of our patients. In our study, we observed AP in 39 (60%) patients in the third trimester, in 17 (26.2%) patients in the second trimester, and in 9 (13.6%) in the first trimester. In a meta-analysis including 823 patients, AP was observed in 64.9% of patients at the third trimester and decreased maternal and fetal mortality as gestational age increased. The highest maternal and fetal mortality was observed in the first trimester, and the lowest prevalence of AP was observed in the first trimester^4,8^.

In a study conducted by Luo et al., which included 121 patients, it was reported that the most common etiology of APDP was biliary stones, followed by hyperlipidemia. Local complications were found to be higher in pancreatitis related to hyperlipidemia. Maternal and fetal mortality rates were correlated with the severity of AP, and they were 3.3% (4/121) and 11.6% (14/121), respectively^4^. In this study, high mortality rates may be related to a high number of pancreatitis cases due to hyperlipidemia. In a study by Tang et al., conducted on 54 patients, despite having no maternal mortality, fetal mortality was found in 11 patients (20.4%). In this study, the most common etiology of pancreatitis was hyperlipidemia and only one patient related to biliary stone had mortality^20^. In our study, only one patient died due to pancreatitis caused by hyperlipidemia. Fetal mortality occurred in the same patient. Only one patient had an early delivery at 37 weeks.

Although previous studies reported high maternal and fetal mortality rates with high undesirable outcomes, nowadays maternal and fetal mortality rates and undesired worse outcomes prevalence are lower. This may be due to patients’ admission to intensive care units in the early period and developments in neonatal intensive care units^5,8^.

Pancreatitis due to hyperlipidemia has worse outcomes than pancreatitis due to other etiologies. The exact mechanism of development of pancreatitis related to hyperlipidemia is unknown^20^. There are several theories of pancreatitis development and its more severe form in hyperlipidemia. High concentrations of chylomicron particles may lead to high blood flow resistance, leading to impairment in pancreatic microcirculation, and even Ischemia and necrosis may occur. Hydrolysis of triglycerides by pancreatic lipase may release free fatty acids, leading to excessive endothelial damage in acinar cells and pancreatic capillaries. At the same time, these free fatty acids may activate trypsinogen, which leads to severe pancreatitis and the activation of severe systemic inflammation. The severity of pancreatitis is correlated with the severity of hypertriglyceridemia^22^. Hyperlipidemia-associated APDP has worse fetal outcomes^20,23^. In pregnant patients, lipid-lowering drugs are contraindicated, and lipid apheresis or plasmapheresis may be done to lower triglyceride levels^23^.

Studies reported that APDP was most observed in the third trimester, and high undesirable outcomes were observed in the first trimester in both fetus and mother^8^. In our study, pancreatitis was observed in the third, second, and first trimesters, respectively. Mortality and undesirable outcomes may be lower due to high number of patients in the third trimester^24^.

In our study, most of our patients had mild pancreatitis according to Atlanta criteria, and Ranson and modified Glasgow scores were low. Most of our patients were treated conservatively. We observed lower maternal or fetal mortality in our patients. We have observed only one maternal mortality and one fetal mortality in the same patient. We thought that lower mortality rates may be related to a high number of mild pancreatitis and a high number of biliary pancreatitis, and most patients were in the third trimester. Besides, early admission of pregnant patients to the hospital and early beginning of proper treatment may also have an important role. Maternal death had occurred in a patient who had a second severe pancreatitis attack related to hyperlipidemia despite lipid apheresis.

Although developments in the treatment of AP in pregnancy and better outcomes have been achieved, AP causes serious stress in patients, their spouses, and relatives. Gastroenterologists and obstetricians must collaborate in the proper treatment and management of APDP for both mother and fetus.

In conclusion, we evaluated pregnant patients with AP, and the most common cause of AP was biliary. Most of the APDP was observed in the third trimester. Most patients had mild pancreatitis. Maternal and fetal complications were rare. We think that the reasons for the low mortality rate were mild disease severity and biliary etiology, and most patients were in the third trimester, as well as early diagnosis and no delay in the intervention.
